# Stratified active archaeal communities in the sediments of Jiulong River estuary, China

**DOI:** 10.3389/fmicb.2012.00311

**Published:** 2012-08-30

**Authors:** Qianqian Li, Fengping Wang, Zhiwei Chen, Xijie Yin, Xiang Xiao

**Affiliations:** ^1^State Key Laboratory of Microbial Metabolism, School of Life Science and BiotechnologyShanghai, P.R. China; ^2^State Key Laboratory of Ocean Engineering, Shanghai Jiao Tong UniversityShanghai, P.R. China; ^3^School of Life Sciences, Xiamen UniversityXiamen, China; ^4^Third Institute of Oceanography, State Oceanic AdministrationXiamen, P.R. China

**Keywords:** archaea, methanogen, ANME, SMTZ, anaerobic oxidation of methane, *mcrA*, estuary, microbial community

## Abstract

Here the composition of total and active archaeal communities in a sediment core of Jiulong River estuary at Fujian Province, Southern China was reported. Profiles of CH_4_ and SO^2−^_4_ concentrations from the sediment core indicated the existence of a sulfate-methane transition zone (SMTZ) in which sulfate reduction-coupled anaerobic oxidation of methane (AOM) occurs. Accordingly, three sediment layers (16–18.5 cm, 71–73.5 cm, and 161–163.5 cm) from the 1.2 m sediment core were sectioned and named top, middle and bottom, respectively. Total DNA and RNA of each layer were extracted and used for clone libraries and sequence analysis of 16S rRNA genes, the reverse transcription (RT)-PCR products of 16S rRNA and methyl CoM reductase alpha subunit (*mcrA*) genes. Phylogenetic analysis indicated that archaeal communities of the three layers were dominated by the Miscellaneous Crenarchaeotal Group (MCG) whose ecological functions were still unknown. The MCG could be further divided into seven subgroups, named MCG-A, B, C, D, E, F, and G. MCG-A and MCG-G were the most active groups in the estuarine sediments. Known anaerobic methanotrophic archaea (ANMEs) were only found as minor components in these estuarine archaeal communities. This study, together with the studies of deep subsurface sediments, would be a very good start point to target and compare the specific active archaeal groups and their roles in the dark, deep subsurface sediment environments.

## Introduction

Marine subsurface sediments probably constitute one of the largest reservoirs of biomass on Earth (Whitman et al., 1998). The diversity of prokaryotic communities in various marine sediments has been studied extensively, but most microbial phylotypes belong to uncultivated groups of unknown physiology and ecological functions (Sørensen and Teske, 2006). Uncultivated archaea, such as Marine Group I (MG-I), Marine Group II (MG-II), Miscellaneous Crenarchaeotic Group (MCG), Marine Benthic Group B (MBGB), Marine Benthic Group D (MBGD) were found not only as dominant groups at some deep-sea sediments, but were also widespread in various environments in nature. These groups were suggested to play important roles in the global cycling of carbon and nitrogen (Orphan et al., 2001; Teske and Sørensen, 2008). However, more investigations were needed to understand the environmental factors associated with their biogeographic distributions, their phylogeny and physiology, and the biogeochemical roles of these archaea in the environment.

Significant amounts of methane are produced in marine sediments. The release of methane to the atmosphere results in the increasing rate of global warming and chemical composition changes (Lelievelda et al., 1993; Hanson and Hanson, 1996). However, nearly all the methane in marine sediments is oxidized before reaching the aerobic waters column and the atmosphere by anaerobic oxidation of methane (AOM) coupled to sulfate reduction catalyzed by microbes in the marine sediments. The main niche for AOM in marine sediments is the sulfate-methane transition zone (SMTZ), where methane produced in the sediments and sulfate from seawater overlap and provide a minimum yield of energy for anaerobic methanotrophs (Knittel and Boetius, 2009). Anaerobic methanotrophic archaea (ANME), named ANME-1, ANME-2, and ANME-3, are believed to be the main players in AOM (Boetius et al., 2000). However, in SMTZs from some deep marine subsurface sediments, such as from Peru Margin sites, ANMEs were not detected, but other uncultivated archaeal groups including South African Gold Mine Euryarchaeotic Group (SAGMEG), MCG and MBGB were found as main components (Inagaki et al., 2006). In a sediment core from Peru Margin site 1227 (Ocean Drilling Program Leg 201), members of MCG and MBGB archaea were found to be more active in the SMTZ than in sediment layers above and below, suggesting either direct or indirect involvement of these archaea in AOM (Sørensen and Teske, 2006).

**Figure A1:**
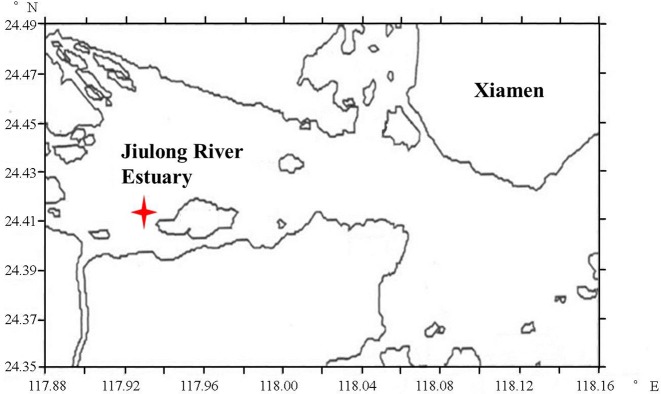
**Map of sampling site in the Jiulong River estuary.** The asterisk indicates the sampling site (24°24′48.6″ N, 117°56′30.5″ E).

Estuarine sediments, with complex geochemical profiles, are another important environment that shows high biological activity rates. The archaeal communities in the sediments of tropical, subtropical and temperate estuaries were dominated by uncultivated archaeal groups, such as MG-I predominant at the near-surface sediments, while MCG distributed throughout the vertical level of the sediment cores (Vieira et al., 2007; Singh et al., 2010; Webster et al., 2010; Jiang et al., 2011). Studies on Pearl River (China) and Santos-Sao Vicente (Brazil) revealed obvious SMTZs in the sediment cores (Saia et al., 2009; Jiang et al., 2011). ANME group ANME-2 was supposed to be the main group with AOM functions in Pearl River estuarine sediment (Jiang et al., 2011). All of these above studies were conducted to investigate the total archaeal community based on the cellular DNA level, which could not exclude the inactive or dead cells persisting in the environments. Here, we aim to investigate the diversity and distribution of both total and active archaeal communities in the sediment of Jiulong River estuary which is located in the southern tropical region in Fujian Province, southern China. The Jiulong River is one of the largest river/estuary systems in southern China with a length of 285 km and an area of 14,741 km^2^ (Maskaoui et al., 2002). The river provides large input of freshwater to the Xiamen's coastal waters (Figure A1). Our study was designed to: (1) reveal the diversity and abundance of archaea in the Jiulong River estuary by 16S rRNA analysis; (2) reveal the active archaeal communities and their distribution along the sediment core; (3) figure out the vertical distribution profile of archaea involved in the methane cycle by functional methyl co-enzyme M reductase A gene (*mcrA*) analysis. This study would provide more information on the distribution and activity of live archaeal communities in estuarine environments, and would be valuable as an analog of deep subsurface habitats in subsurface microbial investigations.

## Materials and methods

### Study site and sampling

The study site is the Jiulong River estuary (24°24′48.6″ N, 117°56′30.5″ E) in Fujian province, China (Figure A1).

A sediment core of 1.2 m was taken using a single-core sampler in December, 2009. The water depth for sampling was about 3.0 m. The bottom water temperature was 13.5°C and the salinity at the sediments surface was 2%. The sediments were mainly composed of sandy clay. The diameter of sediment core was 5.0 cm. The core was sectioned into 2.5 cm slices and transferred to sterile Falcon tubes on a clean bench. Samples were kept at −20°C and then stored at −70°C after back to the laboratory until analysis.

### Methane and sulfate concentration analysis

Methane concentrations were measured as following. The subsamples were immediately taken from the central part of the core. Then, 3.0 ml subsamples were transferred with syringes to Bellco anaerobic tubes (Bellco Glass Inc., Vineland, NJ) each containing 6.0 ml of 1 M NaOH. The vials were closed with black butyl rubber stoppers and aluminum crimp seals immediately. After that, the vials were shaken vigorously for 2 min, and 0.5 ml of gas sample from the headspace of each vial was analyzed by the gas chromatograph (Agilent 6820) equipped with a flame ionization detector using a Porapak Q column (2 m × 3 mm). N_2_ was the carrier gas with a flow rate of 30 ml/min. Methane peaks were recorded and compared with methane standards. The concentration was recalculated to μmol/l pore water using the sediment volume and the independently determined porosity.

The sulfate concentration was determined by ion chromatography (Dionex DX-600) according to the methods of Jiang et al. (2009).

### DNA and RNA extraction and purification

In order to avoid contamination, all the DNA and RNA extractions were carried out using the central part of the sediment core with a diameter of around 1 cm. Three parts of sediment core were separated and labeled as: top (16.0–18.5 cm), middle (71.0–73.5 cm) and bottom (161.0–163.5 cm). The DNA was extracted according to the method described earlier (Xu et al., 2007) and the purification was carried out using a Cycle Pure Kit (OMEGA, USA).

RNA was extracted directly from sediment samples using a Soil RNA Kit according to the manufacturer's manual (OMEGA, USA).

### Clone libraries construction, RFLP analysis, and DNA sequencing

The archaeal 16S rRNA gene fragments were amplified from the three sediment layers by PCR using the primer pair Arch21F (TTCCGGTTGATCCYGCCGGA) and Arch958R (YCCGCGTTGAMTCCAATT) (Lane, 1991; Wagner et al., 1998). The reverse transcription (RT)-PCR of 16S rRNA fragments were carried out using a RevertAid™ First Strand cDNA Synthesis Kit (Fermentas, CAN) by primer Arch958R for the first strand synthesis and PCR amplification by primers Arch21F/Arch958R. For the *mcr*A gene fragments, PCR was performed using the primer pairs ME1 (GCMATGCARATHGGWATGTC) and ME2 (TCATKGCRTAGTTDGGRTAGT) (Hales et al., 1996). The PCR was carried out with the following reaction mix: 100–200 ng sediment DNA, 10.0 pmol of each primer, 10 × PCR reaction buffer, 1.5 mM MgCl_2_, 200.0 μM dNTP, and 5.0 U Taq polymerase, to give a final volume of 50.0 μl. Thermal cycling was performed with the following protocol: 94°C for 4 min, 30 cycles of 94°C for 1 min, 55°C for 1 min, 72°C for 1 min, and a final step at 72°C for 10 min. The negative controls without DNA were set in parallel.

The amplified fragments were purified using UNIQ-10 PCR product purification kit (Sangon). The fragments were ligated into the pMD18-T vector (TaKaRa) following the manufacturer's instructions and transformed into the competent cells of *Escherichia coli* DH5α. Positive clones were randomly picked for Restriction fragment length polymorphisms (RFLP) analysis.

Cloned PCR products were analyzed by RFLP. The PCR products were purified and digested by restriction enzymes *Rsa*I and *Msp*I. The DNA fragments were separated on 3% (w/v) agarose gel by electrophoresis to screen the clones for grouping into similar clone types. Representative clones with unique RFLP bands were chosen for further sequencing using Sanger sequencing method (Sangon Inc., Shanghai, China).

### Quantitative PCR analysis of archaeal 16S rRNA genes

The abundance of archaeal and bacterial 16S rRNA genes was evaluated by fluorescence quantitative real-time PCR with the primer sets Arch344f/Arch519r for archaea (Bano et al., 2004) and Eubac341f/Eubac518r for bacteria (Dilly et al., 2004) on a 7500 Real-time System (Applied Biosystems). Standard curves were constructed by using the method described earlier (Wang et al., 2009). All the amplifications were performed in 20.0 μl reaction mixture with 1.0 μl template DNA, 0.15 μM of each primer, and 10.0 μl of Power SYBR Green PCR Master Mix with ROX and SybrGreen I (Applied Biosystems). Cycle thresholds were set automatically using the 7500 system software, Version 1.3. The average of three replicates was performed.

### Statistical and phylogenetic analysis

The coverage of the library was calculated with the formula C = 1−(n_1_/N), where n_1_ is the number of single-occurrence phylotypes within a library and N is the total number of clones analyzed (Mullins et al., 1995). The Shannon-Wiener index and Evenness (equitability) were calculated using the equations from Krebs (1989). The richness was estimated by Chao1 estimator (http://www2.biology.ualberta.ca/jbrzusto/rarefact.php).

The 16S rRNA genes retrieved in this study were first submitted to the CHIMERA-CHECK program at the Ribosomal Database Project II (Maidak et al., 2001) to check and remove chimeric sequences. The non-chimeric sequences were submitted to the BLAST search program on the NCBI (National Center for Biotechnology Information) website (http://blast.ncbi.nlm.nih.gov/Blast.cgi) and RDP (Ribosomal Database Project) website (http://rdp.cme.msu.edu/) to identify close relatives. The ARB-software package (Ludwig et al., 2004) and SILVA rRNA sequence database (http://www.arb-silva.de/) were used for sequence alignment. Sequences with identities of greater than 97% were tentatively assigned to one OTU (Operation taxonomic units) using the DOTUR (Schloss and Handelsman, 2005). One sequence per OTU was chosen for the construction of phylogenetic trees. The *mcrA* genes were translated into amino acids at SIB ExPASy (Expert Protein Analysis System) website (http://web.expasy.org/translate/). Sequence alignments with portions of both the 16S rRNA gene and deduced amino acids sequences of McrA were carried out by CLUSTAL X 1.83 software. The phylogenetic trees were constructed by the neighbor-joining and minimum evolution method by Mega 3.1 software (Kumar et al., 2004) with the bootstrap analysis used to estimate the confidence of tree topologies (Saitou and Nei, 1987). The phylogenetic trees presented here were constructed by the neighbor-joining method.

### Nucleotide sequence accession numbers

The nucleotide and amino acid sequences obtained in this study were submitted to the NCBI Genbank database with the accession numbers JQ245808–JQ245854 for *mcrA* genes, JQ245855–JQ245893 for RT-PCR products of 16S rRNA and JQ245894–JQ245962 for 16S rRNA genes.

## Results

### Profiles of sulfate and methane

**Figure 1:**
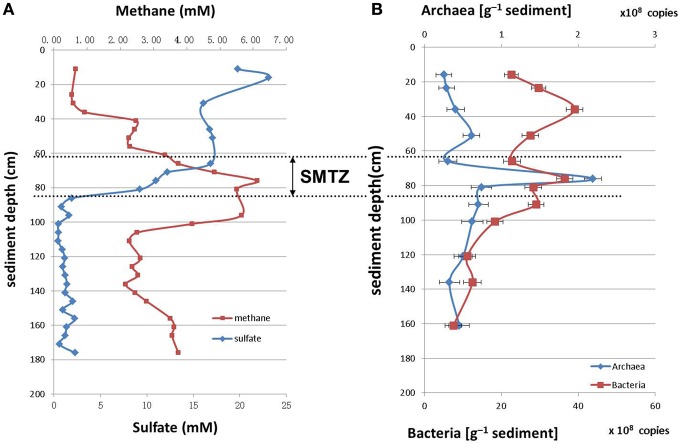
**Depth distributions of methane/sulfate concentrations (A) and archaeal/bacterial 16S rRNA gene abundances (number of gene copies/g [wet weight]) (B) in sediments of Jiulong River estuary**.

The concentrations of sulfate and methane along the sediment core were measured as described in the materials and methods section (Figure 1A). The sulfate concentration was highest at the sediment surface, and declined with the depth to less than 2.0 mM below 86 cm. The methane concentration was low at the sediment surface and increased rapidly within the interval from 56.0 cm to 76.0 cm; highest concentration of 6.0 mM was reached at 76.0 cm depth. Therefore, the depth between 60.0 and 80.0 cm was defined as SMTZ.

### Cell abundance and quantification of archaeal 16S rRNA genes

The archaea and bacteria in the sediment core were quantified by Q-PCR of 16S rRNA genes. The number of bacterial 16S rRNA genes varied from 2.52 × 10^8^ to 2.19 × 10^9^ copies/g (wet weight), and that of archaea were from 10^7^ to 10^8^ copies/g (wet weight) in the sediment core. Overall, the 16S rRNA gene copy number of bacteria was 10 times higher than that of archaea. The archaea reached the highest proportion at the depth between 60.0 and 80.0 cm within the SMTZ (Figure 1B).

### Archaeal community structure

The archaeal communities in the three layers were investigated by library construction and phylogenetic analysis. From each library of the three sediment layers, 50 positive clones were selected randomly for RFLP analysis and sequencing. The coverage values of the 16S rRNA gene libraries were from 85 to 91.5%. According to the Shannon-Wiener index, Simpson's index, Evenness index and Chao-1 estimator, the archaeal diversity in the top layer was higher than the middle and bottom layer (Table 1).

**Table 1:** **Coverage, diversity, and richness evaluation of constructed libraries**.

**Library**	**Layer**	**Coverage %**	**Shannon-Wiener index**	**Simpson's index (1-D)**	**Evenness**	**Chao 1 estimator**
Archaeal 16S rDNA	Top	85.0	3.230	0.957	0.930	33.1
	Middle	87.5	2.997	0.946	0.910	20.8
	Bottom	91.5	2.761	0.930	0.879	19.6
Archaeal 16S rRNA-based	Top	85.2	2.166	0.859	0.793	13.0
	Middle	82.9	1.952	0.774	0.589	18.0
	Bottom	82.9	2.361	0.875	0.707	23.2
*mcrA*	Top	86.3	2.762	0.931	0.879	27.0
	Middle	92.7	2.507	0.910	0.876	15.5
	Bottom	90.5	2.479	0.906	0.852	22.0

**Figure 2:**
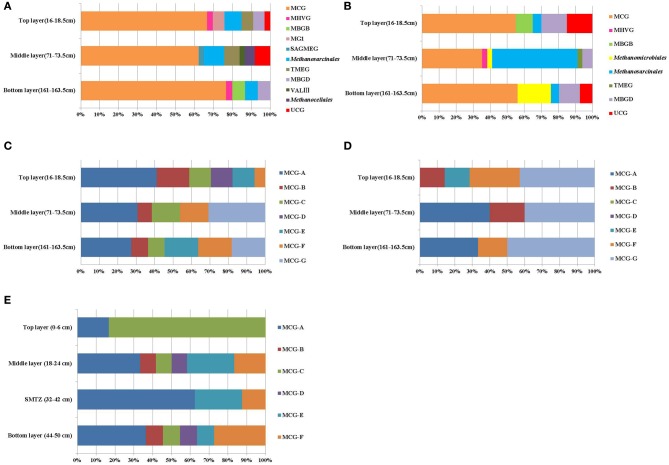
**Compositions of archaeal members in 16S rRNA gene clone libraries (A) and 16S rRNA clone libraries (B), MCG subgroups in the 16S rRNA gene clone libraries (C) and 16S rRNA clone libraries (D), and MCG subgroups in 16S rRNA gene clone libraries of Pearl River Estuary (E).** Groups shown include Miscellaneous Crenarchaeotal Group (MCG), marine benthic group B (MBGB), Marine Group I (MGI), South African Gold Mine Euryarchaeotic Group (SAGMEG), Terrestrial Miscellaneous Euryarchaeotal Group (TMEG), marine benthic group D (MBGD), Lake Valkea Kotinen cluster III (VALIII), Marine Hydrothermal Vent Group (MHVG), *Methanosarcinales*, *Methanomicrobiales*, and *Methanocellales*.

BLAST search results showed that most retrieved archaeal 16S rRNA gene sequences were closely related to uncultured archaeal sequences. Phylogenetic analysis indicated the archaeal communities of the three layers were all composed of *Crenarchaeota* and *Euryarchaeota*. MCG were dominant in all libraries, representing more than 50% of the sequenced clones (Figure 2A). Methanogens within the order *Methanosarcinales* were detected in every layer, most abundantly in the middle layer. However, the ANME groups which had the function of AOM were not detected in the libraries. MG-I was only detected at the top layer; *Methanocellales* and Lake Valkea Kotinen cluster III (VALIII) groups were found in the middle layer; MBGB were detected at bottom layer; Marine Hydrothermal Vent Group (MHVG) and MBGD were represented in both top and bottom layer, but were absent at the middle layer.

**Figure A2:**
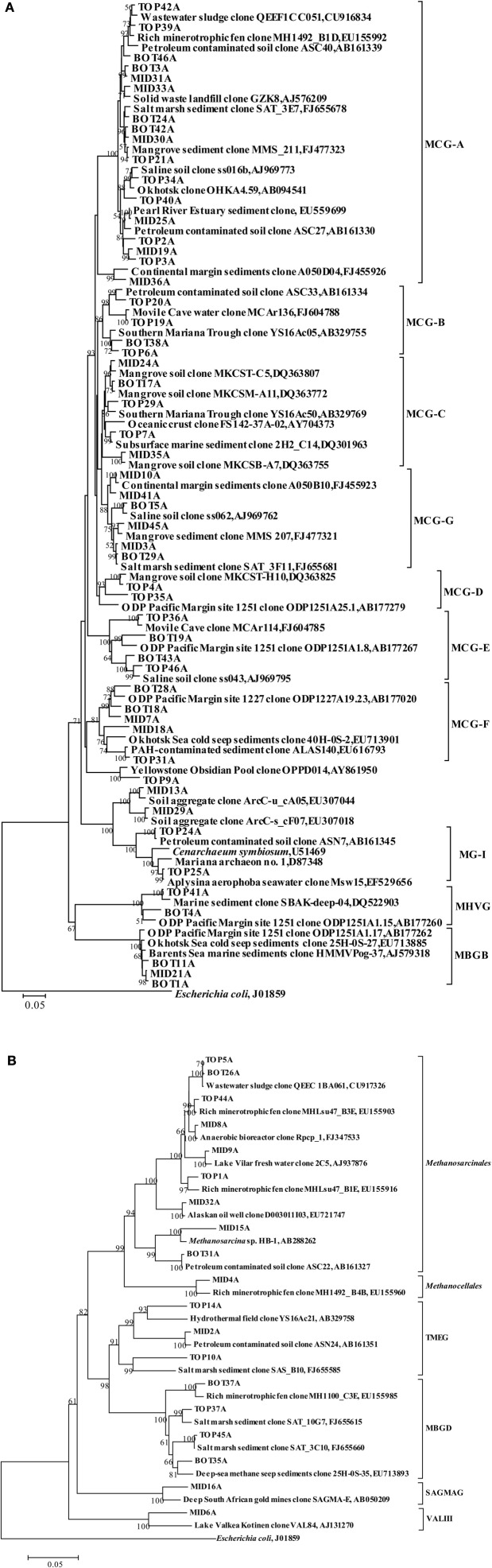
**Phylogenetic trees showing the affiliations of crenarchaeal (A) and euryarchaeal (B) 16S rRNA gene sequences retrieved from this study with selected reference sequences.** The clones from the top, middle, and bottom layers are differentiated as TOP, MID, and BOT, respectively. The trees were constructed by the neighbor-joining, using nearly full-length aligned nucleotides sequences with *E. coli* J01859 as outgroup. Bootstrap values are based on 1000 replicates and are shown at the nodes with more than 50% bootstrap support. The scale bars represent 5% sequence divergence. MCG: Miscellaneous Crenarchaeotal Group; MHVG: Marine Hydrothermal Vent Group; MG1: Marine Group I; SAGMEG: South African Gold Mine Euryarchaeotic Group; MBGB: Marine Benthic Group B; TMEG: Terrestrial Miscellaneous Euryarchaeotal Group; MBGD: Marine Benthic Group D; VALIII: Lake Valkea Kotinen cluster III.

The retrieved crenarchaeal sequences could be classified into MCG, MG-I, and MBGD. Most MCG were closely related to clones from various environments, such as mangrove sediment (GenBank No. FJ477323, DQ363755, DQ363772, and DQ363807), salt marsh sediments (GenBank No. FJ655678 and FJ655681), continental margin sediments (GenBank No. FJ455923 and FJ455926), deep sea sediment with the presence of methane hydrate (GenBank No. EU713901), waste water sludge (GenBank No. CU916834) and petroleum contaminated soil (GenBank No. AB161330, AB161334, and AB161339). According to the previous classification (Jiang et al., 2011), the MCG sequences retrieved from the sediment cores could be assigned to MCG-A, -B, -C, -D, -E, -F, and a new subgroup MCG-G (Figure 2C). Other phylogenetic groups represented only small proportions of the three clone libraries, and these sequences were most closely related to clones from deep sea sediments (Figure A2A).

Sequences within *Methanosarcinales* were most dominant in *Euryarchaeota* (Figure A2B). Related 16S rRNA gene sequences in GenBank originated mostly from wastewater sludge (GenBank No. CU917326), a minerotrophic fen (GenBank No. EU155903 and EU155916), an anaerobic bioreactor (GenBank No. FJ347533), fresh water (GenBank No. AJ937876) and an oil well (GenBank No. EU721747). Clone MID15A was closely related to the cultured species *Methanosarcina horonobensis*, isolated from groundwater in a Miocene subsurface formation (Shimizu et al., 2011). Clones within *Methanocellales*, Terrestrial Miscellaneous Euryarchaeotic Group (TMEG), MBGD, South African Gold Mine Euryarchaeotic Group (SAGMEG) and VALIII were related to clones from a minerotrophic fen (GenBank No. EU155960 and EU155985), a hydrothermal field (GenBank No. AB329758), salt marsh sediments (GenBank No. FJ655585, FJ655615, and FJ655660), and deep-sea methane seep sediments (GenBank No. EU713893).

### Active archaeal community structure

Three 16S rRNA clone libraries were constructed and analyzed in the same way as the 16S rRNA gene clone libraries. The coverage values of the three libraries were from 82.9 to 85.2%. Archaeal diversity in the bottom layer was higher than in the top and middle layer (Table 1).

According to the phylogenetic analysis (Figure 2B), 16S rRNA sequences were affiliated with the MCG, MBGB, and MHVG within *Crenarchaeota*, and with the *Methanosarcinales*, *Methanomicrobiales*, MBGD and TMEG within the *Euryarchaeota*. Most of the archaeal clones were related to uncultivated archaea.

**Figure A3:**
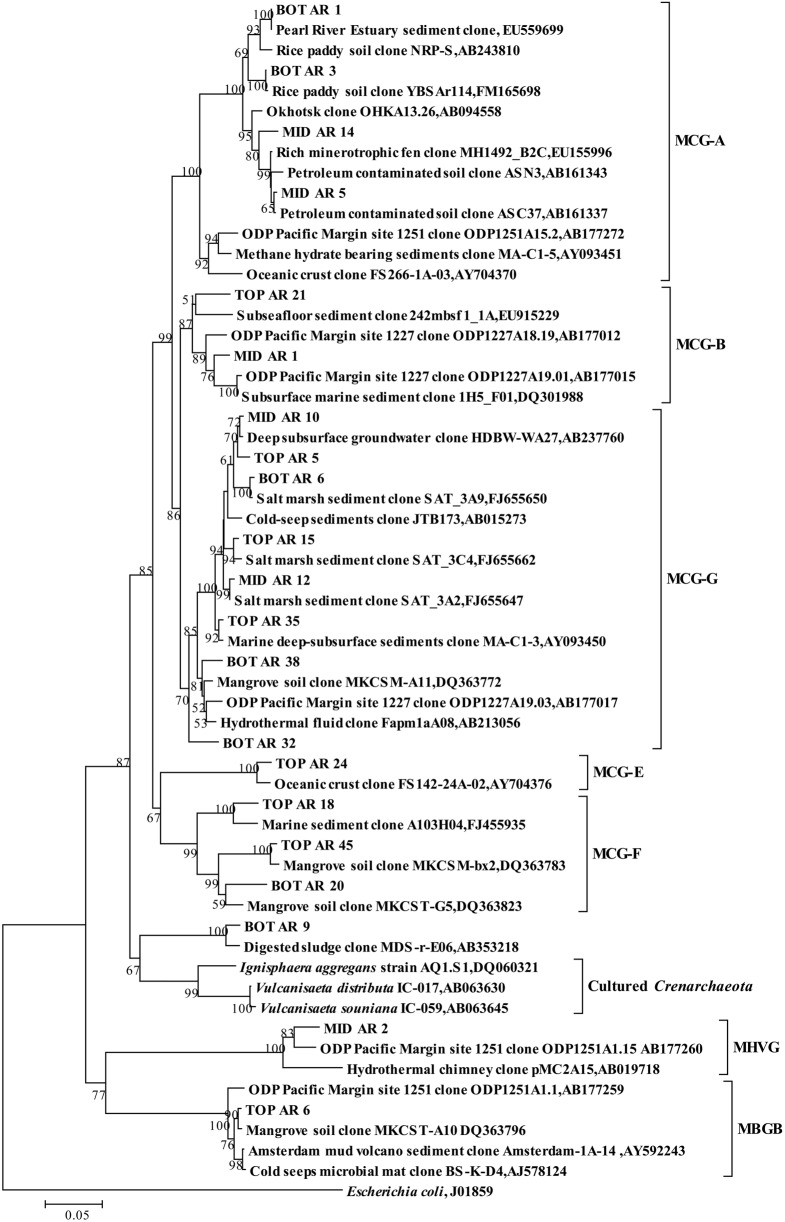
**Phylogenetic tree showing the affiliations of crenarchaeal RT-PCR products of 16S rRNA sequences retrieved from this study with selected reference sequences.** The clones from the top, middle and bottom layers are differentiated as TOP, MID, and BOT, respectively. The tree was constructed by the neighbor-joining, using nearly full-length aligned nucleotides sequences with *E. coli* J01859 as outgroup. Bootstrap values are based on 1000 replicates and are shown at the nodes with more than 50% bootstrap support. The scale bar represents 5% sequence divergence. MCG: Miscellaneous Crenarchaeotal Group; MHVG: Marine Hydrothermal Vent Group; MBGB: Marine Benthic Group B.

The top and bottom layers were dominated by MCG archaea, and specifically by sequences within the MCG-A, MCG-B, MCG-E, MCG-F, and MCG-G subgroups (Figure 2D). Sequences from MCG-G subgroup accounted for 57% of all MCG clones. These sequences were >95% similar to environmental sequences. Sequences of the MCG-A subgroup were detected in middle and bottom layers, while sequences of the MCG-B subgroup were found in the top and middle layers (Figure A3).

**Figure 3:**
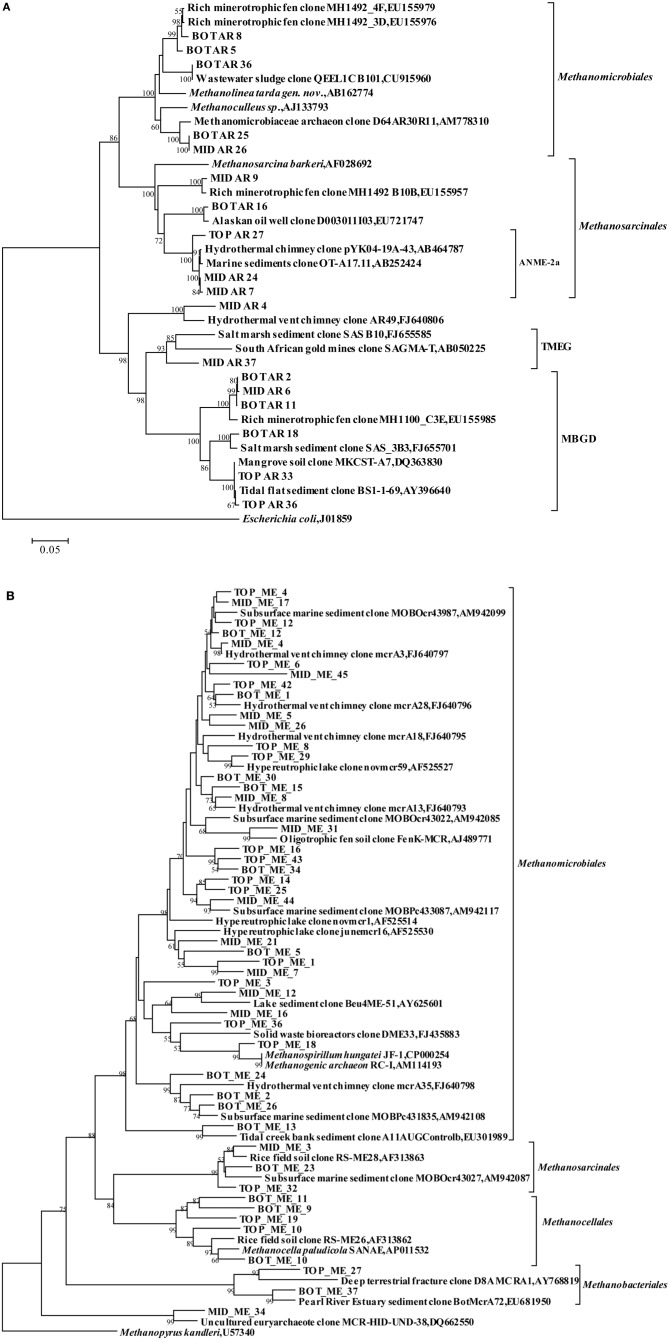
**Phylogenetic trees showing the affiliations of euryarchaeal RT-PCR products of 16S rRNA sequences (A) and *mcrA* gene sequences (B) retrieved from this study with selected reference sequences, respectively.** The clones from the top, middle and bottom layers are differentiated as TOP, MID, and BOT, respectively. The trees were constructed by neighbor-joining, using nearly full-length aligned nucleotides sequences with *Escherichia coli* J01859 or *methanopyrus* as outgroups, respectively. Bootstrap values are based on 1000 replicates and are shown at the nodes with more than 50% bootstrap support. The scale bars represent 5% sequence divergence. TMEG: Terrestrial Miscellaneous Euryarchaeotal Group; MBGD: Marine Benthic Group D; ANME: anaerobic methane-oxidizing archaea.

Among the *Euryarchaeota*, 28% of all clones clustered within the *Methanosarcinales*. Of these, 40% belonged to the ANME-2a branch and originated from the SMTZ. These sequences were closely related to clones from a hydrothermal chimney (GenBank No. AB464787) and marine sediment (GenBank No. AB252424). Clones within the *Methanomicrobiales* and MBGD were also frequently found, and were mostly related to clones from a minerotrophic fen (GenBank No. EU155976, EU155979, and EU155985), salt marsh sediment (GenBank No. FJ655701) and mangrove soil (GenBank No. DQ363830). MBGB was only detected in the top layer (Figure 3A).

### Phylogenetic analysis of *mcrA* genes

Low diversities were found in the three *mcrA* libraries (Table 1), indicating a low diversity of archaea involved in methane cycling in this environment.

**Figure 4:**
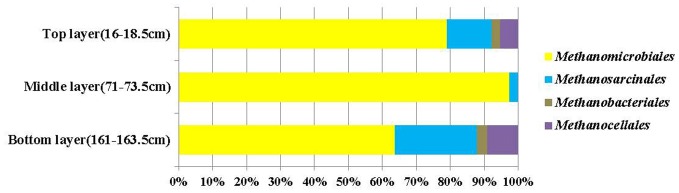
**Compositions of *mcrA* gene clone libraries from top, middle (SMTZ) and bottom layers.** Groups shown include *Methanomicrobiales*, *Methanosarcinales*, *Methanobacteriales*, and *Methanocellales*.

Cloned *mcrA* genes belonged to the *Methanomicrobiales*, *Methanosarcinales*, *Methanobacteriales*, and *Methanocellales*. Members of the *Methanomicrobiales* were predominant, and accounted for an average of 80% in all three clone libraries, whereas members of the *Methanosarcinales*, *Methanobacteriales* and *Methanocellales* constituted around 13, 2, and 5%, respectively (Figure 4). *mcrA* genes from ANME groups were not detected in the libraries.

Among the *Methanomicrobiales*, 44% of all *mcrA* clones from the top, middle and bottom sediment layers showed over 90% similarity with clones from gassy subsurface sediments of Marennes-Oleron Bay and Fuca Ridge hydrothermal vent (GenBank No. AM942085, AM942099, FJ640793, and FJ640795-FJ640798). Only clone MID_ME_45 was 87% similar to clone mcrA3 from Fuca Ridge hydrothermal vent (Wang et al., 2009). Other retrieved sequences of *Methanomicrobiales* shared highest identity with clones from an oligotrophic fen (GenBank No. AJ489771), brackish lake sediment (GenBank No. AY625601), a solid waste bioreactor (GenBank No. FJ435883) and tidal creek sediment (GenBank No. EU301989). Sequences among the order *Methanosarcinales* were closely related (91–98% similarity) to clones from rice field soil (GenBank No. AF313863). Clone BOT_ME_23 was most similar with clones from marine sediment, but also shared 92% sequences similarity with *Methanosarcina horonobensis* (Shimizu et al., 2011). Clones within *Methanocellales* were related to *Methanocella paludicola* (GenBank No. AP011532) with low similarities (84–85%), except clone BOT_ME_10 (95%). Sequences within the *Methanobacteriales* were associated with phylotypes from sediment of the Pearl River Estuary (GenBank No. EU681950) and from deep crustal fluid (GenBank No. AY768819) (Figure 3B).

## Discussion

Most studies on archaeal diversity and distribution were carried out on DNA level, whereas fewer analyses were performed on RNA level to identify active microbial community members (Harrison et al., 2009; Jiang et al., 2011). Here, we investigated not only the abundance and diversity of the archaeal community in the sediments of the Jiulong River estuary, but also the active members by parallel DNA and RNA analysis. Compared with the DNA libraries, the diversity and abundance of clones from RNA-based libraries were lower (Table 1), probably reflecting reduced numbers and phylogenetic diversity of active archaeal community members in the environment. According to the phylogenetic analysis, archaeal members of MCG, MHVG, MBGB, TMEG, and *Methanosarcinales* were common groups in DNA and RNA libraries. On the other hand, MG-I, VALIII, *Methanocellales*, and SAGMEG groups were only found at DNA level, while MBGD, ANME-2, and *Methanomicrobiales* groups were only detected at RNA level. The differences of the archaeal compositions found at the DNA and RNA level suggested a difference of archaeal presence and activity in the environment ((Sørensen and Teske, 2006).

MCG was found to be prevalent through the sediment core. Although MCG was frequently detected in marine and terrestrial environments, the ecological function of this group was still poorly constrained; MCG archaea were suggested to represent heterotrophic anaerobes that utilize and assimilate complex organic substrates (Biddle et al., 2006). Jiang et al divided MCG into six subgroups (MCG-A to MCG-F) (Jiang et al., 2011). We found that MCG-C could be further divided into MCG-C and MCG-G (Figure A2A), and may be further divided into more subgroups when more sequences are available. The MCG-A subgroup was detected most frequently at DNA level, and had the widest distribution among MCG subgroups in all three sediment layers. This result was consistent with the result from that of Pearl River estuarine sediments (Figure 2E) where MCG-A was also identified as the most frequently detected archaeal group (Jiang et al., 2011). The DNA sequences retrieved from the Jiulong River estuary and Pearl River Estuary were both related to similar phylotypes from terrestrial habitats, coastal marine sediments and estuarine sediments. However, although MCG-A was predominant at DNA level, MCG-G subgroup was the most frequently detected at RNA level, and should therefore represent the active archaeal subgroup in this estuarine environment. However, only DNA-level diversity analysis has been carried out in the Pearl River estuary (Jiang et al., 2011), and RNA data from this and other estuaries are still missing. The physiology and ecological function of MCG-G is at present unknown, but it was found widespread in various environments including salt mash sediments, mangrove soil, deep-sea sediments, and hydrothermal fluids (Reed et al., 2002; Yan et al., 2006; Kato et al., 2009; Nelson et al., 2009). The distribution, physiology and biogeochemical functions of different MCG subgroups may vary significantly; more careful and intensive studies are required such as designing of specific primers for specific MCG subgroups to monitor their distribution and the correlation with the environments. Other approaches such as metagenomic, metatranscriptomic analysis, in combination with stable isotope probing and/or single cell sequencing and Nano-SIMS would eventually discover the ecological roles of these unknown uncultivated MCG groups.

Within the *Euryarchaeota*, *Methanosarcinales*, and *Methanocellales* were detected in all layers on DNA level. However, no ANME groups were found. At RNA level, *Methanosarcinales* was the major group, especially in the SMTZ; and *Methanomicrobiales* was the second most dominant methanogenic group. The presence of active methanogens (*Methanosarcinales* and *Methanomicrobiales*) in all three layers indicated methanogenic activity in the sediments. Another Euryarchaeal phylotype detected by 16S rRNA and rDNA analysis was MBGD. Although, MBGD sequences in this study were related to counterparts from various marine and terrestrial environments, the MBGD group is generally associated with methane-rich environments (Pachiadaki et al., 2011). The potential role of MBGD in methane metabolism is still unclear.

ANME groups, known as methane-oxidizing archaea, were not detected at DNA level. ANME-1 is distantly affiliated with the *Methanosarcinales* and *Methanomicrobiales*, while ANME-2 and ANME-3 belong to the *Methanosarcinales* (Hinrichs et al., 1999; Orphan et al., 2001; Knittel et al., 2005). At RNA level, 60% of the clones within the order *Methanosarcinales* could be identified as ANME-2, and 40% of these clones were from the SMTZ. No gene expression of *mcrA* was detected, indicating a low proportion of *mcrA* mRNA in the total RNA sample. Nevertheless, the analysis of *mcrA* gene sequences revealed methanogens in this estuarine environment; phylotypes associated with the *Methanomicrobiales* were found dominant, especially in the middle layer. This divergent *mcrA* and 16S rRNA results might be due to the differences in average copy number between the 16S rRNA and *mcrA* genes in the genomes of different methanogens (Nunoura et al., 2008). However, phylotypes associated with ANME groups were not found in the *mcrA* clone library. In this study, ANME-2 was only detected in the 16S rRNA clone library, but absence in 16S rRNA gene library and *mcrA* library, suggesting a very low proportion of ANME in the community. The flack of detection of ANME phylotypes in the *mcrA* DNA library could also have results from limited clone sequencing performed in this study. It is still an open question whether other archaea detected in this environment such as MBGB, MBGD, and MCG play a role in the methane oxidation process.

Knowing the microbes that are alive or active in the deep subsurface sediment environments will help us to figure out the biogeochemical roles of these species. Our study, together with others, would be a good start to understand the specific live archaeal groups and their roles in the deep subsurface sediment environments. Briefly, this is the first step to reveal the active archaeal members in Jiulong River estuarine sediments, and eventually understand their physiology and biogeochemical roles of these largely unknown uncultivated archaea in nature.

### Conflict of interest statement

The authors declare that the research was conducted in the absence of any commercial or financial relationships that could be construed as a potential conflict of interest.
